# Identification of a Set of Genes Improving Survival Prediction in Kidney Renal Clear Cell Carcinoma through Integrative Reanalysis of Transcriptomic Data

**DOI:** 10.1155/2020/8824717

**Published:** 2020-10-13

**Authors:** Banlai Ruan, Xianzhen Feng, Xueyi Chen, Zhiwei Dong, Qi Wang, Kai Xu, Jinping Tian, Jie Liu, Ziyin Chen, Wenzhen Shi, Man Wang, Lu Qian, Qianshan Ding

**Affiliations:** ^1^Medical Research Center, Xi'an No. 3 Hospital, the Affiliated Hospital of Northwest University, Xi'an, 710016 Shaanxi Province, China; ^2^The College of Life Sciences, Northwest University, Xi'an, 710069 Shaanxi Province, China; ^3^Hubei Yicanhealth Co., Ltd., Wuhan, 430070 Hubei Province, China; ^4^Department of Obstetrics and Gynecology, The Third People's Hospital of Linyi, Linyi, 276000 Shandong Province, China; ^5^Department of Oncology, Affiliated Hospital of Qingdao University, Qingdao, 266000 Shandong Province, China; ^6^Department of Gastroenterology, Renmin Hospital of Wuhan University, Wuhan, 430070 Hubei Province, China

## Abstract

**Background:**

With an enormous amount of research concerning kidney cancer being conducted, various treatments have been applied to its cure. However, high recurrence and metastasis rates continue to pose a threat to the survival of patients with kidney renal clear cell carcinoma (KIRC).

**Methods:**

Data from The Cancer Genome Atlas were downloaded, and a series of analyses were performed, including differential analysis, Cox analysis, weighted gene coexpression network analysis, least absolute shrinkage and selection operator analysis, multivariate Cox analysis, survival analysis, and receiver operating characteristic curve and functional enrichment analysis.

**Results:**

A total of 5,777 differentially expressed genes were identified from the differential analysis. The Cox analysis showed 1,853 significant genes (*P* < 0.01). Weighted gene coexpression network analysis revealed that 226 genes in the module were related to clinical parameters, including Tumor-Node-Metastasis (TNM) staging. Least absolute shrinkage and selection operator and multivariate Cox analyses suggested that four genes (CDKL2, LRFN1, STAT2, and SOWAHB) had a potential function in predicting the survival time of patients with KIRC. Survival analysis uncovered that a high risk of these four genes was associated with an unfavorable prognosis. Receiver operating characteristic curve analysis further confirmed the accuracy of the risk score model. The analysis of clinicopathological parameters of the four identified genes revealed that they were associated with the progression of KIRC.

**Conclusion:**

The gene expression model consisting of CDKL2, LRFN1, STAT2, and SOWAHB is a promising tool for predicting the prognosis of patients with KIRC. The results of this study may provide insights into the diagnosis and treatment of KIRC.

## 1. Introduction

Kidney cancer is one of the most prevalent types of cancer worldwide [1, 2]. Belonging to kidney cancer, kidney renal clear cell carcinoma (KIRC) is characterized by high recurrence and metastasis rates, challenging the health and quality of life of patients [3, 4]. According to statistics, following surgery, the recurrence rate of KIRC may reach 40% [5, 6]. In KIRC, cancer cells often metastasize to other organs [7–9]. In addition, diagnosis of KIRC in the early stage of disease is difficult due to its insidious symptoms. These reasons contribute to the difficulty in treating KIRC.

Bioinformatics analysis has been increasingly important in cancer research for predicting the prognosis of patients and exploring novel therapy targets. Weighted gene coexpression network analysis (WGCNA), least absolute shrinkage and selection operator (LASSO) analysis, and functional enrichment analysis are three of the most popular bioinformatics tools. For example, a recent study identified key pathways and genes in the dynamic progression of hepatocellular carcinoma based on WGCNA [10]. WGCNA may also be applied to construct competing endogenous RNA networks, which are involved in regulating cancer progression [11, 12]. LASSO analysis is often employed to screen the most crucial genes and reduce the number of genes in some models [13]. For instance, a recent study used LASSO analysis to identify prognostic long noncoding RNA signatures in bladder cancer [14]. Functional enrichment analysis is widely utilized in studies to find crucial pathways [15–17].

In this study, mainly using the aforementioned tools, we aimed to establish a model for improving the prediction of survival of patients with KIRC. With data from The Cancer Genome Atlas (TCGA), after obtaining differentially expressed genes (DEGs), Cox analysis was performed to preliminarily detect prognosis-related genes. Subsequently, WGCNA was used to set up a gene coexpression network, and LASSO analysis was employed to delete highly correlated genes, and multivariate Cox analysis was utilized to construct a survival prediction model. We found that a panel of four genes, including cyclin-dependent kinase like 2 (CDKL2), leucine-rich repeat and fibronectin type III domain-containing 1 (LRFN1), signal transducer and activator of transcription 2 (STAT2), and sosondowah ankyrin repeat domain family member B (SOWAHB), was a promising module for predicting the survival of patients with KIRC. Subsequently, functional enrichment analysis was performed to analyze the biological events regulated by this module.

## 2. Materials and Methods

### 2.1. Data Acquisition and Processing

RNA sequencing data of KIRC samples (72 normal samples and 538 tumor samples) and relevant clinical information of patients with KIRC were downloaded from TCGA (https://portal.gdc.cancer.gov/). Survival information of 530 samples was available, and the details of the patients are presented in Table 1. Data regarding disease-free survival were downloaded from cBioPortal (http://www.cbioportal.org/). In the process of constructing a risk model, 530 samples were divided into two groups using the R package caret (265 in the training and testing groups, respectively) (Table S1).

### 2.2. Identification of DEGs

The function package edgeR was utilized to conduct a differential analysis. We selected ∣Log‐fold change | >1 and false discovery rate < 0.05 as significant cutoff values based on the Benjamini–Hochberg method. A heat map was generated to show the expression levels of genes in normal and tumor samples.

### 2.3. WGCNA

WGCNA was performed to combine significant prognostic DEGs with clinical traits [18]. The function hclust was used to cluster samples and delete outliers. The soft-thresholding power was chosen based on the criterion of approximate scale-free topology after the function pickSoftThreshold was performed. According to the soft-thresholding power *β*, a weighted gene network with a relatively large minimum module size of 30 was constructed. The parameter mergeCutHeight was the threshold to merge of modules. Next, the modules that were significantly associated with the clinical traits were identified. Subsequently, the correlation between modules and clinical traits was determined. The associations of individual genes with clinical traits were quantified by defining gene significance (GS) as the correlation between genes and clinical traits. For each module, the quantitative measure of module membership (MM) was treated as the correlation of the module eigengene and the gene expression profile. GS and MM were highly correlated, illustrating that genes significantly associated with a trait were often also the most important (central) elements of modules related to the trait. Based on this, genes highly significantly associated with clinical traits could be identified.

### 2.4. Construction of a Cox Model

LASSO analysis and multivariate Cox regression analysis were conducted to construct a risk model. The 226 significant prognosis genes (blue module) identified through these analyses were ranked according to their *P* values. The top 30 significant prognostic genes were calculated by LASSO analysis. After deleting high correlation genes, a multivariate Cox analysis was performed. *P* values < 0.05 indicated statistical significance. The hazard ratio and 95% confidence interval for each variable were calculated.

### 2.5. Functional Enrichment Analysis

Functional enrichment analysis included Gene Ontology (GO) analysis and Kyoto Encyclopedia of Genes and Genomes (KEGG) analysis. GO and KEGG analyses were carried out using the R package clusterProfiler. GO analysis contained biological process, cellular component, and molecular function. *P* < 0.05 indicated statistical significance.

### 2.6. Correlation between Clinical Traits and CDKL2, LRFN1, STAT2, and SOWAHB

Correlation between the four genes and clinical parameters (Tumor-Node-Metastasis (TNM) stage, pathological stage, and grade) was analyzed to further confirm the importance of the identified genes. *P* < 0.05 indicated statistical significance.

### 2.7. Survival Analysis

Online survival analysis and relapse-free survival (RFS) analysis were performed through Gene Expression Profiling Interactive Analysis (GEPIA; http://gepia.cancer-pku.cn/index.html) to recognize significant prognostic biomarkers. *P* < 0.05 indicated statistical significance. Moreover, survival analysis of the risk model was performed using R package survival, and receiver operating characteristic (ROC) curve was constructed based on the R package survival ROC.

## 3. Results

### 3.1. DEGs in KIRC Samples

**Figure 1:**
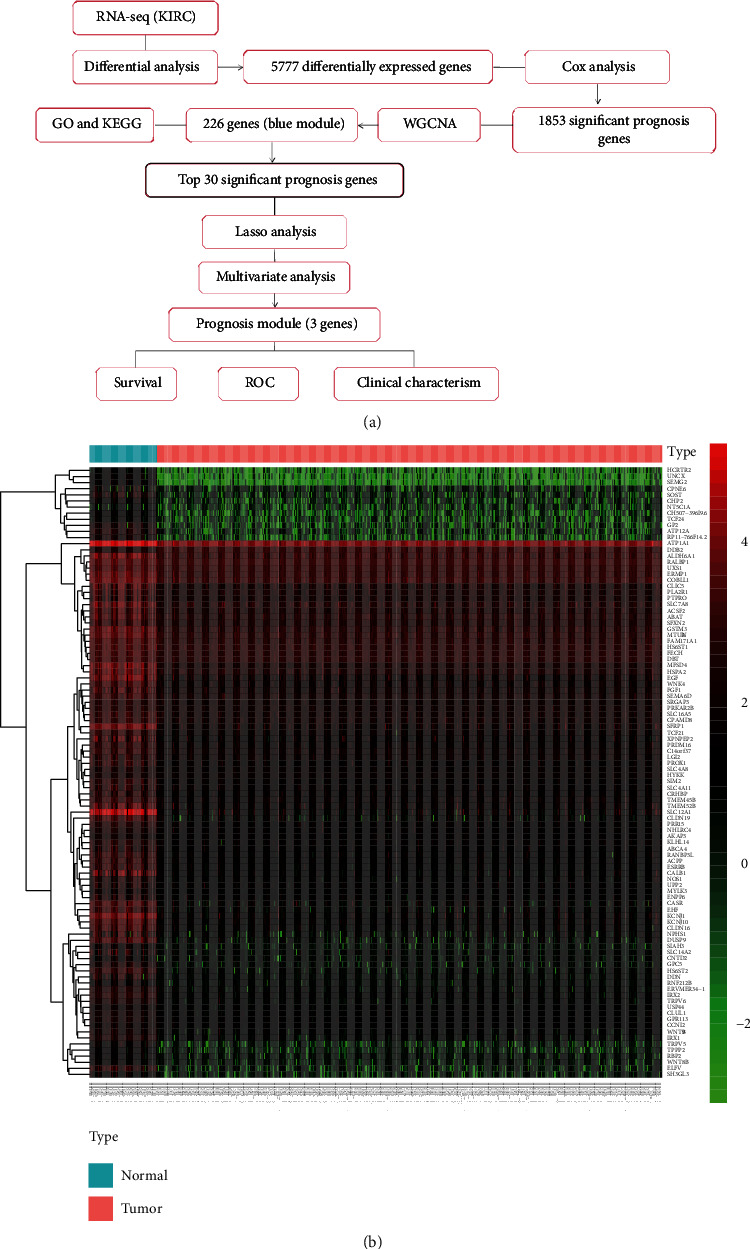
Flowchart of this work and expression of DEGs in TCGA data. (a) KIRC: kidney renal clear cell carcinoma; GO: Gene Ontology; KEGG: Kyoto Encyclopedia of Genes and Genomes; WGCNA: weighted gene coexpression network analysis; LASSO: least absolute shrinkage and selection operator; ROC: receiver operating characteristic curve. (b) Heat map of DEGs. From green to black to red, the expression of gene increased. The blue panels represented normal samples; the red panels represented tumor samples. DEGs: differentially expressed genes.

The workflow is shown in Figure 1(a). RNA sequencing data of KIRC samples were processed by edgeR, and 5,777 DEGs (3,913 upregulation and 1,863 downregulation) were obtained. The heat map of the top 100 DEGs represents the expression level of DEGs in normal tissues and tumor tissues (Figure 1(b)). From the heat map, a significant difference in the expression levels of genes between normal tissues and tumor tissues was observed. Subsequently, univariate Cox analysis was performed, and 1,853 genes significantly related to the prognosis of patients were identified (data not shown).

### 3.2. Results of WGCNA

**Figure 2:**
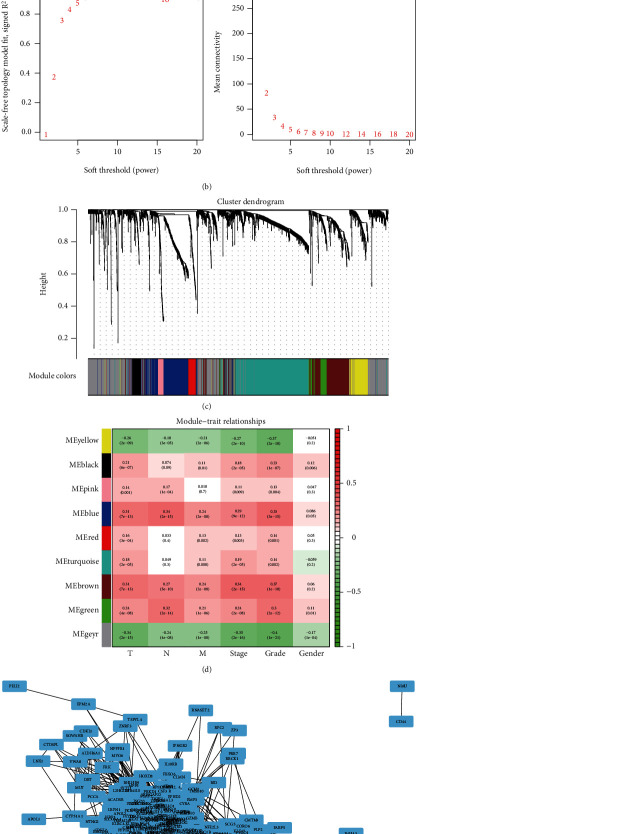
The results of weighted gene coexpression network analysis. (a) Sample clustering was employed to detect outliers. (b) Analysis of network topology for various soft-thresholding powers. The left panel showed that the scale-free fit index (*y*-axis) served as a function of the soft-thresholding power (*x*-axis). The right panel displayed that the mean connectivity (degree, *y*-axis) worked as a function of the soft-thresholding power (*x*-axis). (c) Clustering dendrogram of genes with dissimilarity was based on topological overlap and assigned module colors. (d) Associations between module and trait. Each small square contained the corresponding correlation coefficient and *P* value. (e) Gene coexpression network of the genes in the blue module.

After performing hclust, two samples (TCGA-B0-4696-01 and TCGA-BP-4770-01) were deleted (Figure 2(a)). According to scale independence and mean connectivity, the soft-thresholding power *β* = 6 was considered to be the fittest value, which was responsible for high correlation and high connectivity between genes (Figure 2(b)). Consistent with the thresholding power, these DEGs were divided into eight effective gene modules, and the grey module was considered an ineffective module for preserving nonmodular genes (Figure 2(c)). Through the correlation between GS and MM, we noted the blue module, in which genes were related to TNM staging and tumor grade. As shown in Figure 2(d), blue module genes were highly connected with clinical traits. Correlation between the blue module and T was 0.31, *P* = 7*e* − 13; correlation between the blue module and N was 0.34, *P* = 2*e* − 15; correlation between the blue module and M was 0.24, *P* = 2*e* − 08; correlation between the blue module and clinical stage was 0.29, *P* = 9*e* − 12; and correlation between the blue module and tumor grade was 0.33, *P* = 3*e* − 15. In addition, Cytoscape (https://cytoscape.org/download.html) was used to construct a gene coexpression network based on blue module genes (Figure 2(e)). From the gene coexpression network, we observed that most genes exhibited a strong correlation.

### 3.3. Construction of the Gene Risk-Score System

**Figure 3:**
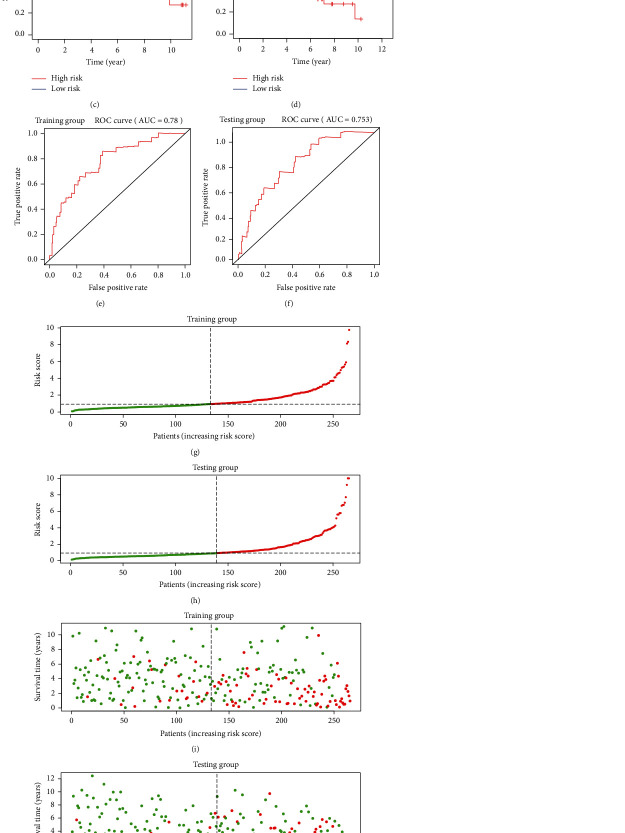
Construction of the gene risk-score system. (a, b) Selection of relative coefficients of the gene risk-score system. (c, d) Survival of the gene risk-score system. (e, f) ROC curve of the gene risk-score system. (g–j) Survival time and status of high-/low-risk groups. (k, l) Expression of four genes (STAT2, LRFN1, CDKL2, and SOWAHB) in high-/low-risk groups. From green to black to red, the expression of gene increased. The red color of the type represented the low-risk group, and blue color of the type represented the high-risk group.

To construct a risk-score system, we selected the top 30 genes from the blue module which were deemed to be the most significant genes according to their *P* value (Table 2). Through LASSO analysis and multivariate Cox regression analysis, a gene risk-score system was obtained using relative coefficients (Figures 3(a) and 3(b)). Subsequently, RELT TNF receptor (RELT), transmembrane protein 245 (TMEM245), receptor accessory protein 4 (REEP4), leucine-rich repeat and fibronectin type III domain-containing 1 (LRFN1), and vesicle-associated membrane protein 1 (VAMP1) were excluded, and the final risk score formula was as follows: PI = (−0.23946 × expression level of CDKL2) + (0.58372 × expression level of STAT2) + (−0.12572 × expression level of SOWAHB) + (0.25274 × expression level of LRFN1). Among these genes, CDKL2 and SOWAHB had negative coefficients in the univariate and multivariate Cox regression analyses, suggesting that upregulating their expression levels would improve the survival time of patients with KIRC. According to the risk score, we divided patients into high- and low-risk groups. In both the training and testing groups, the 5-year survival rate in the high- and low-risk groups was 40% and 80%, respectively (Figures 3(c) and 3(d)). The ROC curve analysis further confirmed the accuracy of the risk-score model, and the area under the curve was 0.78 and 0.753 in the training and testing groups, respectively. After dividing 530 patients into the high-/low-risk groups in the training and testing groups, the risk scores of the patients were negatively associated with the patients' survival time (Figures 3(g)–3(j)). The heat map suggested that STAT2 and LRFN1 were high-risk genes, whereas CDKL2 and SOWAHB were low-risk genes (Figures 3(k) and 3(l)). These results suggested that STAT2, LRFN1, CDKL2, and SOWAHB were prognosis-related genes, and the aforementioned formula could be used to assess the risk of death in patients.

### 3.4. Correlations between the Four Genes and Clinical Traits

**Figure 4:**
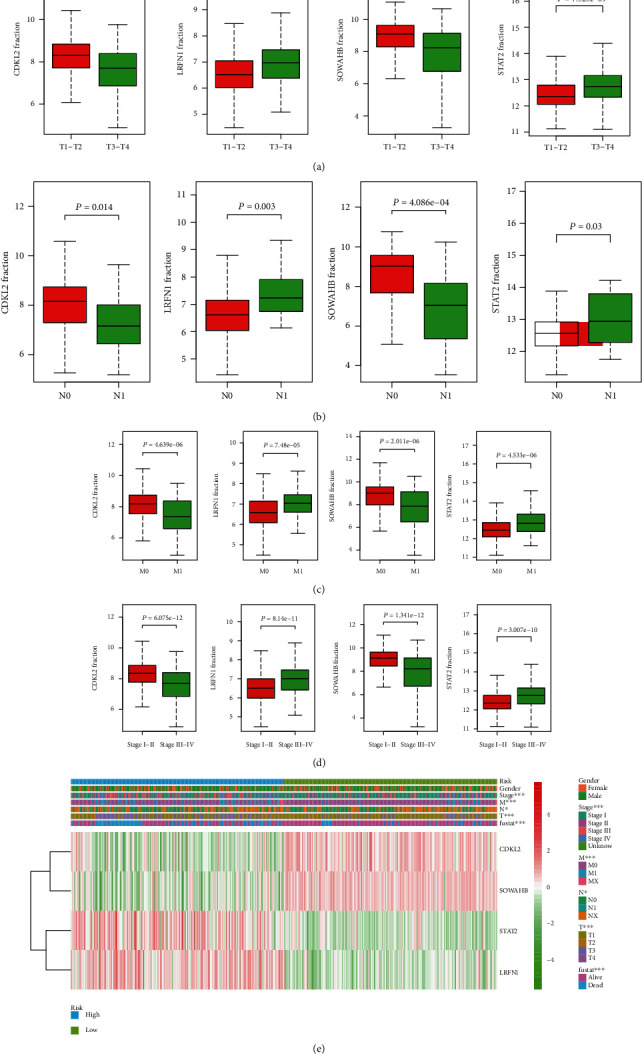
Correlation between clinical traits and genes. (a) Expressions of four genes (STAT2, LRFN1, CDKL2, and SOWAHB) in T1/2 and T3/4 tumors. T: tumor. (b) Expressions of four genes in tumors with/without lymphatic metastasis. N: lymphatic node. (c) Expressions of four genes in tumors with/without distant metastasis. M: distant metastasis. (d) Expressions of four genes in stage I/II and stage III/IV tumors. (e) Correlation between risk model and clinical traits.

**Figure 5:**
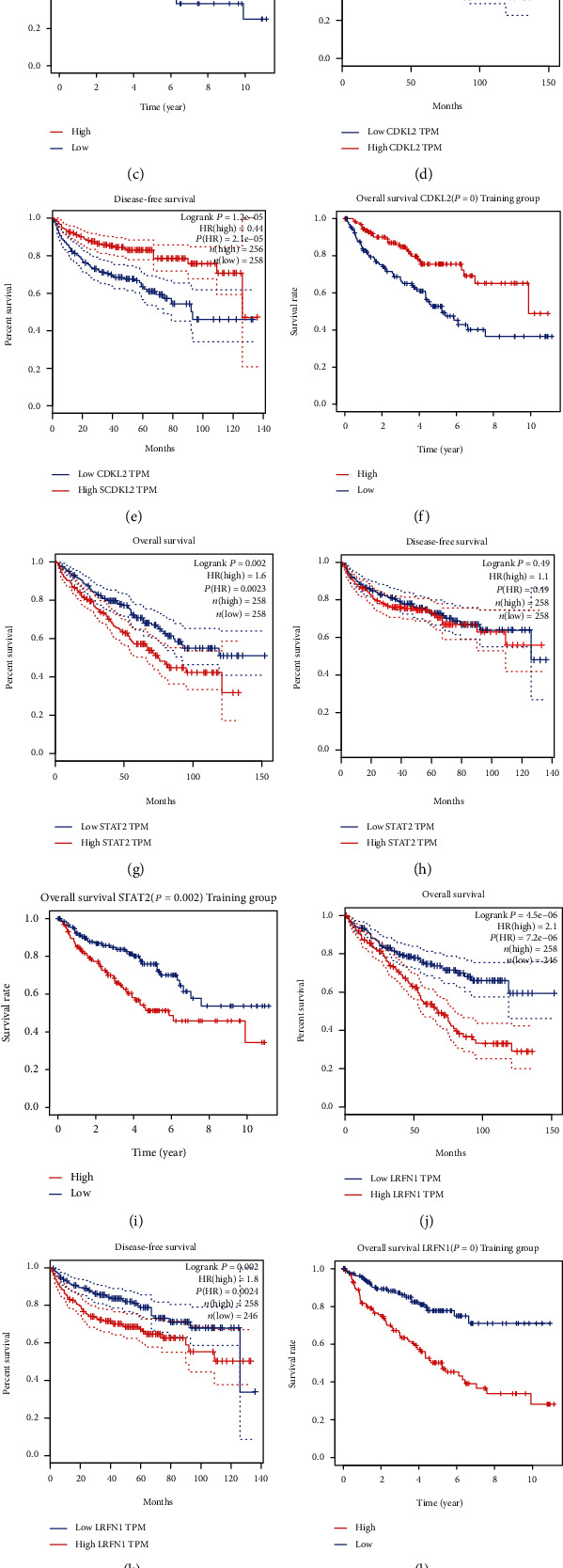
Survival analysis of four genes (STAT2, LRFN1, CDKL2, and SOWAHB). (a, b) OS and RFS plot of SOWAHB by GEPIA. (c) OS plot of SOWAHB in the training group. (d, e) OS and RFS plot of CDKL2 by GEPIA. (f) OS plot of CDKL2 in the training group. (g, h) OS and RFS plot of STAT2 by GEPIA. (i) OS plot of STAT2 in the training group. (j, k) OS and RFS plot of LRFN1 by GEPIA. (l) OS plot of LRFN1 in the training group. (m, n) RFS of risk model in the training group and the testing group. OS: overall survival; RFS: disease-free survival.

The associations between genes and clinical traits (T stage, N stage, M status, clinical stage, tumor grade, etc.) were analyzed to further clarify the clinical importance of the four identified genes. The results showed that CDKL2 and SOWAHB had lower expression levels, while LRFN1 and STAT2 had higher expression levels in T3/4 tumors versus T1/2 tumors (Figure 4(a)), tumors with lymphatic metastasis (Figure 4(b)), tumors with distant metastasis (Figure 4(c)), and stage III/IV tumors versus stage I/II tumors (Figure 4(d)). In addition, we found that the risk model was also related to TNM, staging, and survival status (Figure 4(e)). To verify these results, we performed correlation analysis using an online website (https://mexpress.be/index.html). The findings showed that these four genes were related to numerous clinical traits (Figure S1). These results suggested that STAT2, LRFN1, CDKL2, and SOWAHB may have an impact on the progression, invasion, and metastasis of KIRC. Additionally, online overall survival (OS) and RFS analyses were performed to further confirm the prognostic value of these genes. The results indicated that high expression levels of STAT2 and LRFN1 were associated with poor prognosis of patients with KIRC; in contrast, high expression levels of CDKL2 and SOWAHB indicated favorable prognosis among patients with KIRC (Figures 5(a)–5(i)). Additionally, the risk model showed that RFS of patients with high risk was shorter compared with that in the low-risk group, in both the training and testing groups (Figures 5(m) and 5(n)).

### 3.5. GO and KEGG Pathway Analysis

**Figure 6:**
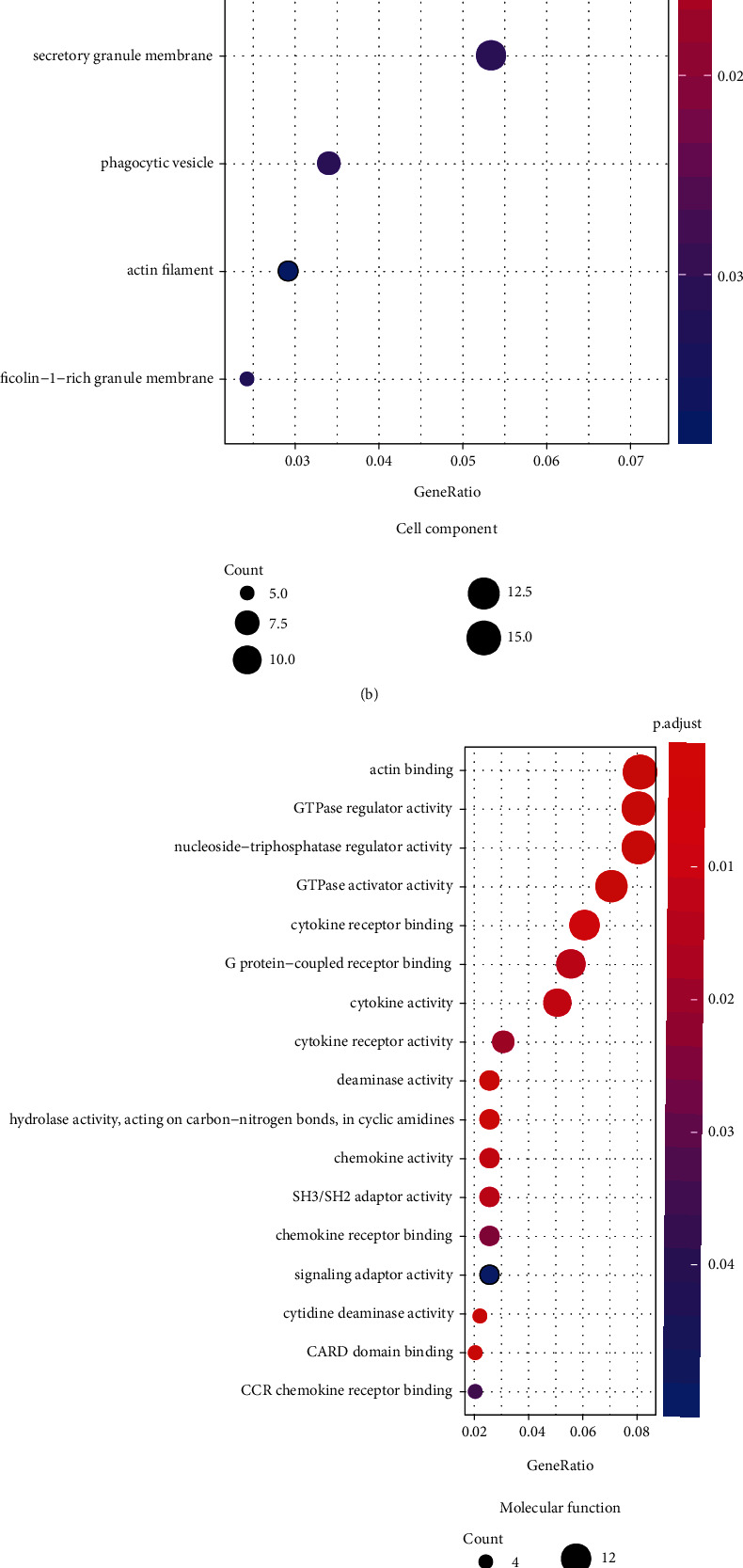
Results of functional enrichment analysis. (a) The top 20 of biological processes potentially regulated by the genes in the blue module. (b) Cell components potentially modulated by the genes in the blue module. (c) Molecular function potentially modulated by the genes in the blue module. (d) Signal pathways potentially regulated by the genes in the blue module. From purple to red, the *P* value decreased. The size of the dots represented gene numbers.

We performed functional enrichment analysis with the R package clusterProfiler to investigate the function and pathway potentially regulated by the genes in the blue module. These genes were mainly enriched in the following: biological process (including T cell activation, regulation of T cell activation, regulation of lymphocyte activation, response to virus, and regulation of cell-cell adhesion (Figure 6(a)); cellular component (including actin cytoskeleton, endocytic vesicle, secretory granule membrane, phagocytic vesicle, and ficolin-1-rich granule membrane (Figure 6(b)); and molecular function, (including actin binding, GTPase regulator activity, nucleoside-triphosphatase regulator activity, GTPase activator activity, and G protein-coupled receptor binding (Figure 6(c))). The pathways potentially regulated by these genes were related to the NOD-like receptor signaling pathway, cytokine-cytokine receptor interaction, osteoclast differentiation, viral protein interaction with cytokine and cytokine receptor, JAK-STAT signaling pathway, T helper 17 cell differentiation, etc. (Figure 6(d)). These results suggested that genes in the blue module were involved in regulating the progression of KIRC via these pathways.

## 4. Discussion

Comprehensive analysis of the gene expression signature in cancer tissues is of great significance in cancer research. It benefits the diagnosis of cancer and provides novel therapeutic targets for its treatment. In the field of KIRC research, depending on the open source gene expression profile data, several pilot studies apply bioinformatics analysis to construct prognosis prediction models or screen hub genes in cancer progression. For instance, using WGCNA and a protein-protein interaction network, a recent study analyzed the gene expression pattern of 26 pairs of tumor tissues/adjacent tissues and identified four hub genes involved in the progression of KIRC, including AGXT, PTGER3, SLC12A3, and ALOX5 [19]. Another study used LASSO and best subset regression to detect prognostic genes in KIRC from TCGA data, including PADI1, ATP6V0D2, DPP6, C9orf135, and PLG [20]. Furthermore, through TCGA data, another research group screened key splicing factors regulating the alternative splicing events during the tumorigenesis of KIRC, which helps elucidate the mechanism of KIRC progression [21]. High-throughput technologies, such as gene expression chip and RNA sequencing, provide a considerable amount of data to researchers. Optimization of the workflow and combination of multiple analysis methods will provide novel clues. This study presented a novel model for predicting the prognosis of patients with KIRC. Functional enrichment analysis suggested that the genes involved in this model were crucial modulators in the progression of KIRC. Additionally, this model only consisted of four genes, concise and precise, showing a preferable application perspective.

Prognosis factors are important indicators of disease treatment [22, 23]. Cox regression analysis is an effective tool to find out prognosis factors [24]. Cox regression analysis includes univariate Cox analysis and multivariate Cox analysis [25]. Moreover, univariate Cox analysis is usually used to screen potential prognosis factors, and multivariate Cox analysis is frequently applied to construct prognosis models [22, 26–30]. In our study, a risk model was constructed based on Cox regression analysis, which benefits the prognostic evaluation and personalized medicine for patients with KIRC. In our risk model, among the four identified genes, CDKL2 and SOWAHB were protective factors for patients with KIRC, whereas LRFN1 and STAT2 were risk factors for these patients. Belonging to the STAT family, STAT2 is a well-characterized oncogene and a crucial component of the interferon- (IFN-) *α*/*β*/*γ* signaling pathway. Together with STAT1 and IRF9, STAT2 forms the IFN-stimulated gene factor 3 (ISGF3) complex and translocates into the nuclei to trigger the transcription of target genes after activation [31]. Moreover, STAT2 is highly expressed or abnormally activated in multiple types of cancer and promotes malignant biological behaviors, including the proliferation, migration, invasion, and epithelial-to-mesenchymal transition of cancer cells [31–33]. However, the expression characteristics, biological function, and underlying mechanism of STAT2 in KIRC have not been systemically investigated. Interestingly, a recent study reported that the IFN-*γ* signaling pathway is significantly activated in renal cancer patients with metastatic disease [34]. The results of that study suggested that STAT2 may participate in the progression of KIRC, which is consistent with our finding in the present study. It is worth investigating the role of STAT2 and STAT2-related pathways in the progression of KIRC in future studies.

The function of CDKL2 in different types of cancer is distinct. It functions as an oncogene in breast cancer to facilitate the process of epithelial-to-mesenchymal transition, inducing the expression of zinc finger E-box-binding homeobox 1 (ZEB1) and promoting the conversion of CD24^high^ cells to CD44^high^ cancer cells [35]. However, in gliomas, hepatocellular carcinoma, and gastric cancer, its underexpression or hypermethylation of its promoter indicates poor prognosis of patients; additionally, overexpression of CDKL2 in gastric cancer cells suppresses the growth and invasion of cancer cells [36–38]. These results suggest that CDKL2 is a tumor suppressor in these types of cancer. Previously, there was no report on the role of CDKL2 in KIRC. Herein, our data implied that downregulation of CDKL2 in KIRC tissues indicated poor prognosis of patients. It is possible that CDKL2 functions as a tumor suppressor in KIRC; however, this hypothesis requires further investigation through *in vitro* and *in vivo* studies.

Importantly, the model utilized in the present study identified two rarely investigated genes, namely, LRFN1 and SOWAHB. A genome-wide association study suggested that SOWAHB was associated with the susceptibility of chronic obstructive pulmonary disease [39]. The biological function of SOWAHB in cancer biology remains obscure. A previous study showed that LRFN1 belongs to the SALM/LRFN family and is a neuronal component in the developing of mature vertebrate nervous system [40, 41]. Our data suggest that LRFN1 and SOWAHB are potential regulators in KIRC progression. Therefore, it is desirable to investigate their biological functions in the following studies.

## 5. Conclusion

In summary, we utilized a comprehensive analysis to construct a novel risk-score model for KIRC, by which we can predict the prognosis of patients. Our results provide potential biomarkers and therapeutic targets, which may be beneficial for the diagnosis and treatment of KIRC.

## Figures and Tables

**Table 1:** Clinicopathological features.

Clinicopathological parameters	Frequency	Percentage
Gender		
Male	346	64.43%
Female	191	35.57%
Pathologic stage		
I-II	326	60.71%
III-IV	208	38.73%
Unknown	3	0.56%
T stage		
T1-T2	344	64.06%
T3-T4	193	35.94%
N stage		
N0	240	44.69%
N1	17	3.17%
NX	280	52.14%
M stage		
M0	446	83.05%
M1	81	15.08%
MX	10	1.86%
Age		
<60	247	46.00%
≥60	290	54.00%

**Table 2:** Univariate Cox regression analysis of the top 30 genes in the blue module.

Gene	HR	HR, 95 CI (low)	HR, 95 CI (high)	*P* value
CHFR	1.000833824	1.000626465	1.001041226	3.18*E* − 15
STAT2	1.000139807	1.000104508	1.000175106	8.28*E* − 15
RELT	1.00168529	1.00123172	1.002139065	3.17*E* − 13
LRFN1	1.003620135	1.002620544	1.004620723	1.18*E* − 12
REEP4	1.000911177	1.000655593	1.001166827	2.75*E* − 12
VAMP1	1.00096664	1.000690669	1.001242688	6.53*E* − 12
TCIRG1	1.000176371	1.000125551	1.000227193	1.03*E* − 11
SOWAHB	0.998170856	0.997638284	0.998703712	1.77*E* − 11
C17orf62	1.000290856	1.000205244	1.000376475	2.75*E* − 11
IGFLR1	1.002196092	1.001540895	1.002851717	4.88*E* − 11
STAC3	1.002374184	1.001648701	1.003100193	1.37*E* − 10
FKBP11	1.000228649	1.000158225	1.000299078	1.97*E* − 10
HAPLN3	1.000541992	1.000373872	1.000710141	2.62*E* − 10
MICAL1	1.000230074	1.000158663	1.000301489	2.70*E* − 10
SH3BGRL3	1.000044229	1.000030246	1.000058211	5.66*E* − 10
CASP4	1.000282876	1.000188726	1.000377036	3.88*E* − 09
CDKL2	0.997021707	0.99601962	0.998024803	6.12*E* − 09
IFI30	1.001967645	1.001302802	1.002632928	6.46*E* − 09
NOD2	1.001225892	1.00081042	1.001641536	7.23*E* − 09
TMEM245	0.999832818	0.999775898	0.99988974	8.61*E* − 09
MPP5	0.999453585	0.999267007	0.999640198	9.59*E* − 09
IL15RA	1.000470293	1.000309107	1.000631504	1.07*E* − 08
FCGR1B	1.004084857	1.002680825	1.005490855	1.13*E* − 08
ARHGEF1	1.000150458	1.000098375	1.000202543	1.49*E* − 08
RNF166	1.000630491	1.00041224	1.000848789	1.49*E* − 08
ACADSB	0.99962285	0.999491681	0.999754037	1.76*E* − 08
UNC13D	1.000234229	1.000152484	1.00031598	1.95*E* − 08
PPP1R18	1.000179058	1.000116469	1.00024165	2.05*E* − 08
MYO6	0.999791047	0.99971764	0.999864459	2.43*E* − 08
RHBDF2	1.000227139	1.000147305	1.00030698	2.45*E* − 08

Abbreviations: HR: hazard ratio; CI: confidence interval.

## Data Availability

The data used to support the findings of this study are available from the corresponding author upon request.

## Supplementary Materials

Supplementary 1 Supplementary Figure S1 Association between 4 genes, including STAT2, LRFN1, CDKL2, and SOWAHB, and various clinical features in the KIRC cohort from TCGA. Statistics *P* ≥ 0.05, ^∗^*P* < 0.05, ^∗∗^*P* < 0.01, and ^∗∗∗^*P* < 0.001; *r* representing coefficient index when analyzing continuous parameters. KIRC: kidney renal clear cell carcinoma; TCGA: The Cancer Genome Atlas.

Supplementary 2 Supplementary Table S1: sample information of training and testing groups from 530 KIRC samples. KIRC: kidney renal clear cell carcinoma.
