# Supplementary figures and images for: Identification of a Set of Genes Improving Survival Prediction in Kidney Renal Clear Cell Carcinoma through Integrative Reanalysis of Transcriptomic Data
Source: Dis Markers. 2020 Oct 13;2020:8824717. doi: 10.1155/2020/8824717 (PMC7578724; doi:10.1155/2020/8824717)

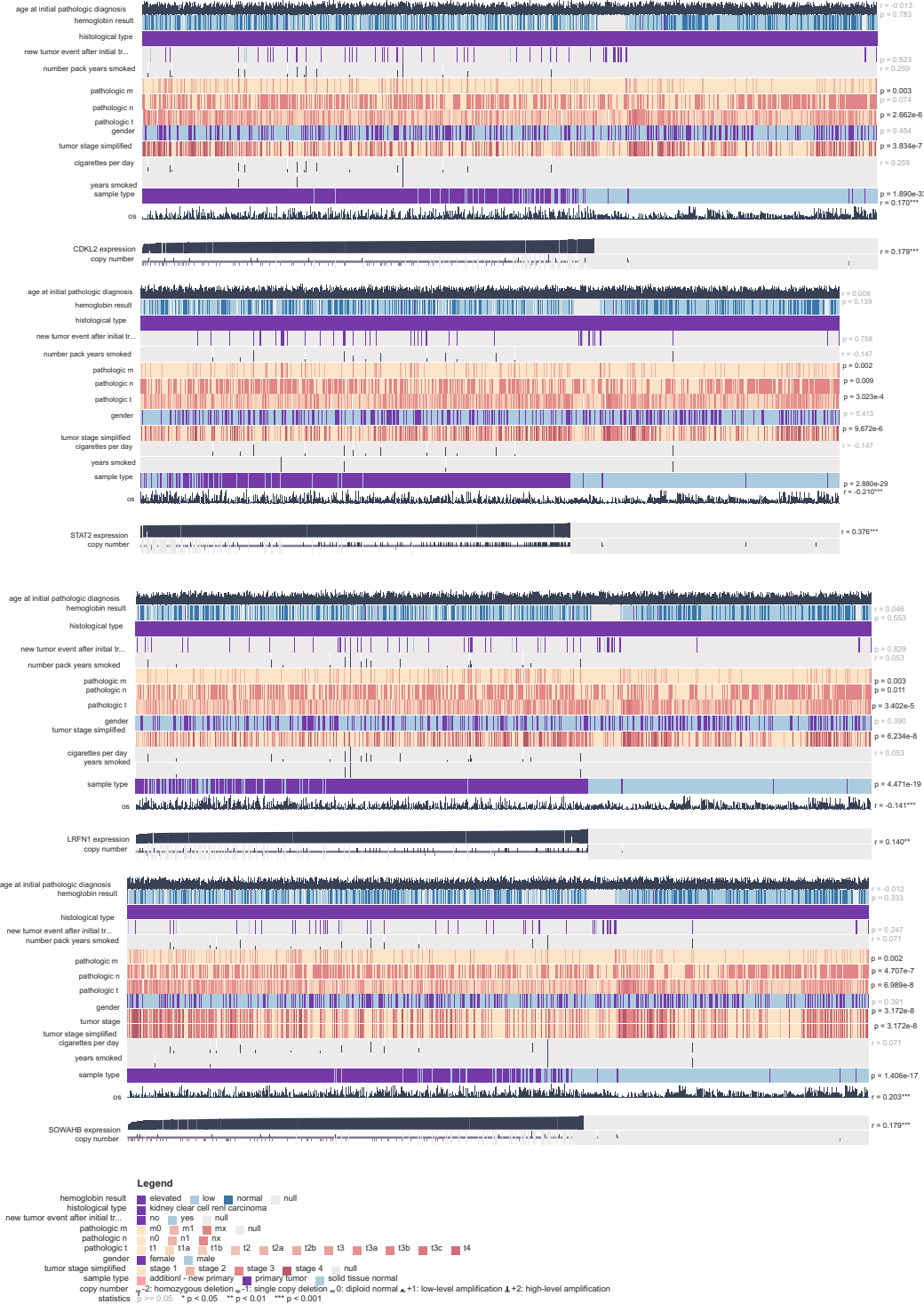

Supplement: Supplementary 1 — Supplementary Figure S1 Association between 4 genes, including STAT2, LRFN1, CDKL2, and SOWAHB, and various clinical features in the KIRC cohort from TCGA. Statistics P ≥ 0.05, ∗P < 0.05, ∗∗P < 0.01, and ∗∗∗P < 0.001; r representing coefficient index when analyzing continuous parameters. KIRC: kidney renal clear cell carcinoma; TCGA: The Cancer Genome Atlas. [file 8824717.f1.zip › Figure S1.pdf]
