# Supplementary material for: Identification of a Set of Genes Improving Survival Prediction in Kidney Renal Clear Cell Carcinoma through Integrative Reanalysis of Transcriptomic Data
Source: Dis Markers. 2020 Oct 13;2020:8824717. doi: 10.1155/2020/8824717 (PMC7578724; doi:10.1155/2020/8824717)
Supplement: Supplementary 2 — Supplementary Table S1: sample information of training and testing groups from 530 KIRC samples. KIRC: kidney renal clear cell carcinoma. [file 8824717.f2.docx]

| Train group | Test group |
| --- | --- |
| TCGA-6D-AA2E  TCGA-A3-3311  TCGA-A3-3313  TCGA-A3-3319  TCGA-A3-3324  TCGA-A3-3326  TCGA-A3-3328  TCGA-A3-3329  TCGA-A3-3331  TCGA-A3-3335  TCGA-A3-3351  TCGA-A3-3352  TCGA-A3-3359  TCGA-A3-3363  TCGA-A3-3365  TCGA-A3-3370  TCGA-A3-3374  TCGA-A3-3376  TCGA-A3-3378  TCGA-A3-3385  TCGA-A3-3387  TCGA-A3-A6NJ  TCGA-A3-A8CQ  TCGA-AK-3425  TCGA-AK-3427  TCGA-AK-3428  TCGA-AK-3429  TCGA-AK-3434  TCGA-AK-3436  TCGA-AK-3445  TCGA-AK-3447  TCGA-AK-3450  TCGA-AK-3456  TCGA-AK-3461  TCGA-AK-3465  TCGA-AS-3778  TCGA-B0-4690  TCGA-B0-4691  TCGA-B0-4699  TCGA-B0-4701  TCGA-B0-4703  TCGA-B0-4707  TCGA-B0-4712  TCGA-B0-4718  TCGA-B0-4810  TCGA-B0-4811  TCGA-B0-4815  TCGA-B0-4818  TCGA-B0-4819  TCGA-B0-4822  TCGA-B0-4823  TCGA-B0-4828  TCGA-B0-4836  TCGA-B0-4837  TCGA-B0-4838  TCGA-B0-4841  TCGA-B0-4843  TCGA-B0-4847  TCGA-B0-4945  TCGA-B0-5075  TCGA-B0-5077  TCGA-B0-5081  TCGA-B0-5084  TCGA-B0-5092  TCGA-B0-5094  TCGA-B0-5095  TCGA-B0-5098  TCGA-B0-5102  TCGA-B0-5108  TCGA-B0-5109  TCGA-B0-5117  TCGA-B0-5119  TCGA-B0-5120  TCGA-B0-5121  TCGA-B0-5399  TCGA-B0-5400  TCGA-B0-5402  TCGA-B0-5692  TCGA-B0-5696  TCGA-B0-5699  TCGA-B0-5700  TCGA-B0-5701  TCGA-B0-5709  TCGA-B0-5711  TCGA-B0-5713  TCGA-B0-5812  TCGA-B2-3923  TCGA-B2-3924  TCGA-B2-4101  TCGA-B2-4102  TCGA-B2-5633  TCGA-B2-5636  TCGA-B2-5639  TCGA-B2-A4SR  TCGA-B4-5377  TCGA-B4-5835  TCGA-B4-5843  TCGA-B8-4153  TCGA-B8-4154  TCGA-B8-4619  TCGA-B8-4620  TCGA-B8-4621  TCGA-B8-4622  TCGA-B8-5158  TCGA-B8-5162  TCGA-B8-5164  TCGA-B8-5165  TCGA-B8-5546  TCGA-B8-5550  TCGA-B8-5553  TCGA-B8-A54D  TCGA-B8-A54F  TCGA-B8-A54I  TCGA-B8-A54J  TCGA-B8-A54K  TCGA-BP-4158  TCGA-BP-4160  TCGA-BP-4161  TCGA-BP-4162  TCGA-BP-4165  TCGA-BP-4167  TCGA-BP-4170  TCGA-BP-4173  TCGA-BP-4174  TCGA-BP-4176  TCGA-BP-4329  TCGA-BP-4341  TCGA-BP-4343  TCGA-BP-4344  TCGA-BP-4345  TCGA-BP-4346  TCGA-BP-4347  TCGA-BP-4349  TCGA-BP-4351  TCGA-BP-4352  TCGA-BP-4353  TCGA-BP-4355  TCGA-BP-4756  TCGA-BP-4759  TCGA-BP-4760  TCGA-BP-4763  TCGA-BP-4765  TCGA-BP-4768  TCGA-BP-4769  TCGA-BP-4770  TCGA-BP-4771  TCGA-BP-4775  TCGA-BP-4776  TCGA-BP-4781  TCGA-BP-4789  TCGA-BP-4799  TCGA-BP-4803  TCGA-BP-4961  TCGA-BP-4963  TCGA-BP-4964  TCGA-BP-4968  TCGA-BP-4969  TCGA-BP-4970  TCGA-BP-4972  TCGA-BP-4974  TCGA-BP-4975  TCGA-BP-4976  TCGA-BP-4977  TCGA-BP-4981  TCGA-BP-4985  TCGA-BP-4986  TCGA-BP-4989  TCGA-BP-5001  TCGA-BP-5006  TCGA-BP-5007  TCGA-BP-5008  TCGA-BP-5009  TCGA-BP-5168  TCGA-BP-5169  TCGA-BP-5173  TCGA-BP-5174  TCGA-BP-5176  TCGA-BP-5177  TCGA-BP-5178  TCGA-BP-5194  TCGA-BP-5196  TCGA-BP-5199  TCGA-BP-5200  TCGA-CJ-4634  TCGA-CJ-4635  TCGA-CJ-4636  TCGA-CJ-4637  TCGA-CJ-4638  TCGA-CJ-4641  TCGA-CJ-4644  TCGA-CJ-4871  TCGA-CJ-4874  TCGA-CJ-4876  TCGA-CJ-4882  TCGA-CJ-4884  TCGA-CJ-4887  TCGA-CJ-4890  TCGA-CJ-4891  TCGA-CJ-4892  TCGA-CJ-4893  TCGA-CJ-4894  TCGA-CJ-4897  TCGA-CJ-4899  TCGA-CJ-4902  TCGA-CJ-4903  TCGA-CJ-4905  TCGA-CJ-4912  TCGA-CJ-4918  TCGA-CJ-5671  TCGA-CJ-5672  TCGA-CJ-5675  TCGA-CJ-5676  TCGA-CJ-5677  TCGA-CJ-5679  TCGA-CJ-5681  TCGA-CJ-5683  TCGA-CJ-5684  TCGA-CJ-6027  TCGA-CJ-6030  TCGA-CJ-6031  TCGA-CJ-6033  TCGA-CW-5583  TCGA-CW-5584  TCGA-CW-5585  TCGA-CW-5588  TCGA-CW-5590  TCGA-CW-5591  TCGA-CW-6087  TCGA-CW-6093  TCGA-CW-6097  TCGA-CZ-4853  TCGA-CZ-4857  TCGA-CZ-4859  TCGA-CZ-4860  TCGA-CZ-4861  TCGA-CZ-4862  TCGA-CZ-4863  TCGA-CZ-4864  TCGA-CZ-4865  TCGA-CZ-5452  TCGA-CZ-5453  TCGA-CZ-5461  TCGA-CZ-5462  TCGA-CZ-5463  TCGA-CZ-5464  TCGA-CZ-5465  TCGA-CZ-5467  TCGA-CZ-5984  TCGA-CZ-5989  TCGA-DV-5565  TCGA-DV-5566  TCGA-DV-5567  TCGA-DV-5568  TCGA-DV-5569  TCGA-DV-5575  TCGA-DV-A4VX  TCGA-DV-A4VZ  TCGA-DV-A4W0  TCGA-EU-5904  TCGA-EU-5905  TCGA-EU-5906  TCGA-G6-A8L6  TCGA-G6-A8L7  TCGA-MM-A564  TCGA-T7-A92I | \| TCGA-3Z-A93Z \| \| --- \| \| TCGA-A3-3306 \| \| TCGA-A3-3307 \| \| TCGA-A3-3308 \| \| TCGA-A3-3316 \| \| TCGA-A3-3317 \| \| TCGA-A3-3320 \| \| TCGA-A3-3322 \| \| TCGA-A3-3323 \| \| TCGA-A3-3325 \| \| TCGA-A3-3343 \| \| TCGA-A3-3346 \| \| TCGA-A3-3347 \| \| TCGA-A3-3349 \| \| TCGA-A3-3357 \| \| TCGA-A3-3358 \| \| TCGA-A3-3362 \| \| TCGA-A3-3367 \| \| TCGA-A3-3372 \| \| TCGA-A3-3373 \| \| TCGA-A3-3380 \| \| TCGA-A3-3382 \| \| TCGA-A3-3383 \| \| TCGA-A3-A6NI \| \| TCGA-A3-A6NL \| \| TCGA-A3-A6NN \| \| TCGA-A3-A8OU \| \| TCGA-A3-A8OV \| \| TCGA-A3-A8OW \| \| TCGA-A3-A8OX \| \| TCGA-AK-3426 \| \| TCGA-AK-3431 \| \| TCGA-AK-3433 \| \| TCGA-AK-3440 \| \| TCGA-AK-3443 \| \| TCGA-AK-3451 \| \| TCGA-AK-3453 \| \| TCGA-AK-3454 \| \| TCGA-AK-3455 \| \| TCGA-AK-3458 \| \| TCGA-AK-3460 \| \| TCGA-AS-3777 \| \| TCGA-B0-4688 \| \| TCGA-B0-4693 \| \| TCGA-B0-4694 \| \| TCGA-B0-4696 \| \| TCGA-B0-4697 \| \| TCGA-B0-4698 \| \| TCGA-B0-4700 \| \| TCGA-B0-4706 \| \| TCGA-B0-4710 \| \| TCGA-B0-4713 \| \| TCGA-B0-4714 \| \| TCGA-B0-4813 \| \| TCGA-B0-4814 \| \| TCGA-B0-4816 \| \| TCGA-B0-4817 \| \| TCGA-B0-4821 \| \| TCGA-B0-4824 \| \| TCGA-B0-4827 \| \| TCGA-B0-4833 \| \| TCGA-B0-4834 \| \| TCGA-B0-4839 \| \| TCGA-B0-4842 \| \| TCGA-B0-4844 \| \| TCGA-B0-4845 \| \| TCGA-B0-4846 \| \| TCGA-B0-4848 \| \| TCGA-B0-4849 \| \| TCGA-B0-4852 \| \| TCGA-B0-5080 \| \| TCGA-B0-5083 \| \| TCGA-B0-5085 \| \| TCGA-B0-5088 \| \| TCGA-B0-5096 \| \| TCGA-B0-5097 \| \| TCGA-B0-5099 \| \| TCGA-B0-5100 \| \| TCGA-B0-5104 \| \| TCGA-B0-5106 \| \| TCGA-B0-5107 \| \| TCGA-B0-5110 \| \| TCGA-B0-5113 \| \| TCGA-B0-5115 \| \| TCGA-B0-5116 \| \| TCGA-B0-5690 \| \| TCGA-B0-5691 \| \| TCGA-B0-5693 \| \| TCGA-B0-5694 \| \| TCGA-B0-5695 \| \| TCGA-B0-5697 \| \| TCGA-B0-5698 \| \| TCGA-B0-5702 \| \| TCGA-B0-5703 \| \| TCGA-B0-5705 \| \| TCGA-B0-5706 \| \| TCGA-B0-5707 \| \| TCGA-B0-5710 \| \| TCGA-B0-5712 \| \| TCGA-B2-4098 \| \| TCGA-B2-4099 \| \| TCGA-B2-5635 \| \| TCGA-B2-5641 \| \| TCGA-B4-5378 \| \| TCGA-B4-5832 \| \| TCGA-B4-5834 \| \| TCGA-B4-5836 \| \| TCGA-B4-5838 \| \| TCGA-B4-5844 \| \| TCGA-B8-4143 \| \| TCGA-B8-4146 \| \| TCGA-B8-4148 \| \| TCGA-B8-4151 \| \| TCGA-B8-5159 \| \| TCGA-B8-5163 \| \| TCGA-B8-5545 \| \| TCGA-B8-5549 \| \| TCGA-B8-5551 \| \| TCGA-B8-5552 \| \| TCGA-B8-A54E \| \| TCGA-B8-A54G \| \| TCGA-B8-A54H \| \| TCGA-B8-A7U6 \| \| TCGA-B8-A8YJ \| \| TCGA-BP-4159 \| \| TCGA-BP-4163 \| \| TCGA-BP-4164 \| \| TCGA-BP-4166 \| \| TCGA-BP-4169 \| \| TCGA-BP-4177 \| \| TCGA-BP-4325 \| \| TCGA-BP-4326 \| \| TCGA-BP-4327 \| \| TCGA-BP-4330 \| \| TCGA-BP-4331 \| \| TCGA-BP-4332 \| \| TCGA-BP-4334 \| \| TCGA-BP-4335 \| \| TCGA-BP-4337 \| \| TCGA-BP-4338 \| \| TCGA-BP-4340 \| \| TCGA-BP-4342 \| \| TCGA-BP-4354 \| \| TCGA-BP-4758 \| \| TCGA-BP-4761 \| \| TCGA-BP-4762 \| \| TCGA-BP-4766 \| \| TCGA-BP-4774 \| \| TCGA-BP-4777 \| \| TCGA-BP-4782 \| \| TCGA-BP-4784 \| \| TCGA-BP-4787 \| \| TCGA-BP-4790 \| \| TCGA-BP-4795 \| \| TCGA-BP-4797 \| \| TCGA-BP-4798 \| \| TCGA-BP-4801 \| \| TCGA-BP-4804 \| \| TCGA-BP-4807 \| \| TCGA-BP-4959 \| \| TCGA-BP-4960 \| \| TCGA-BP-4962 \| \| TCGA-BP-4965 \| \| TCGA-BP-4967 \| \| TCGA-BP-4971 \| \| TCGA-BP-4973 \| \| TCGA-BP-4982 \| \| TCGA-BP-4983 \| \| TCGA-BP-4987 \| \| TCGA-BP-4991 \| \| TCGA-BP-4992 \| \| TCGA-BP-4993 \| \| TCGA-BP-4994 \| \| TCGA-BP-4995 \| \| TCGA-BP-4998 \| \| TCGA-BP-4999 \| \| TCGA-BP-5000 \| \| TCGA-BP-5004 \| \| TCGA-BP-5010 \| \| TCGA-BP-5170 \| \| TCGA-BP-5175 \| \| TCGA-BP-5180 \| \| TCGA-BP-5181 \| \| TCGA-BP-5182 \| \| TCGA-BP-5183 \| \| TCGA-BP-5184 \| \| TCGA-BP-5185 \| \| TCGA-BP-5186 \| \| TCGA-BP-5187 \| \| TCGA-BP-5189 \| \| TCGA-BP-5190 \| \| TCGA-BP-5191 \| \| TCGA-BP-5192 \| \| TCGA-BP-5195 \| \| TCGA-BP-5198 \| \| TCGA-BP-5201 \| \| TCGA-BP-5202 \| \| TCGA-CJ-4639 \| \| TCGA-CJ-4640 \| \| TCGA-CJ-4642 \| \| TCGA-CJ-4643 \| \| TCGA-CJ-4868 \| \| TCGA-CJ-4869 \| \| TCGA-CJ-4870 \| \| TCGA-CJ-4872 \| \| TCGA-CJ-4873 \| \| TCGA-CJ-4875 \| \| TCGA-CJ-4878 \| \| TCGA-CJ-4881 \| \| TCGA-CJ-4885 \| \| TCGA-CJ-4886 \| \| TCGA-CJ-4888 \| \| TCGA-CJ-4889 \| \| TCGA-CJ-4895 \| \| TCGA-CJ-4900 \| \| TCGA-CJ-4901 \| \| TCGA-CJ-4904 \| \| TCGA-CJ-4907 \| \| TCGA-CJ-4908 \| \| TCGA-CJ-4916 \| \| TCGA-CJ-4920 \| \| TCGA-CJ-5678 \| \| TCGA-CJ-5680 \| \| TCGA-CJ-5682 \| \| TCGA-CJ-5686 \| \| TCGA-CJ-5689 \| \| TCGA-CJ-6028 \| \| TCGA-CJ-6032 \| \| TCGA-CW-5580 \| \| TCGA-CW-5581 \| \| TCGA-CW-5587 \| \| TCGA-CW-5589 \| \| TCGA-CW-6088 \| \| TCGA-CW-6090 \| \| TCGA-CZ-4854 \| \| TCGA-CZ-4856 \| \| TCGA-CZ-4858 \| \| TCGA-CZ-4866 \| \| TCGA-CZ-5451 \| \| TCGA-CZ-5454 \| \| TCGA-CZ-5455 \| \| TCGA-CZ-5456 \| \| TCGA-CZ-5457 \| \| TCGA-CZ-5458 \| \| TCGA-CZ-5459 \| \| TCGA-CZ-5460 \| \| TCGA-CZ-5466 \| \| TCGA-CZ-5468 \| \| TCGA-CZ-5469 \| \| TCGA-CZ-5470 \| \| TCGA-CZ-5982 \| \| TCGA-CZ-5985 \| \| TCGA-CZ-5986 \| \| TCGA-CZ-5987 \| \| TCGA-CZ-5988 \| \| TCGA-DV-5573 \| \| TCGA-DV-5574 \| \| TCGA-DV-5576 \| \| TCGA-EU-5907 \| \| TCGA-G6-A5PC \| \| TCGA-G6-A8L8 \| \| TCGA-GK-A6C7 \| \| TCGA-MM-A563 \| \| TCGA-MM-A84U \| \| TCGA-MW-A4EC \| |
